# Correlations between periodontal disease, mandibular inferior cortex index and the osteoporotic fracture probability assessed by means of the fracture risk assessment body mass index tool

**DOI:** 10.1186/s12880-019-0337-1

**Published:** 2019-05-22

**Authors:** Paweł Kalinowski, Ingrid Różyło-Kalinowska, Magdalena Piskórz, Urszula Bojakowska-Komsta

**Affiliations:** 1 Independent Epidemiology Unit, Medical University of Lublin, Lublin, Poland; 2 Department of Dental and Maxillofacial Radiology, Medical University of Lublin, 20–059 Lublin, ul. Karmelicka 7 Poland

**Keywords:** Osteoporosis, Mandible, Alveolar bone loss, FRAX

## Abstract

**Background:**

The aim was to examine correlations between radiological signs of chronic periodontitis, Mandibular Inferior Cortex (MIC) index and osteoporotic fracture probability based on the FRAX BMI tool.

**Methods:**

The material comprised 422 panoramic radiographs taken in patients aged 40–89, 270 females and 152 males. The severity of chronic periodontitis and resorption of mandibular inferior cortex based on MIC index were assessed. A diagnostic survey was conducted to estimate 10-year major and hip osteoporotic fracture probability (MOFP, HOFP) by means of the FRAX BMI tool - an algorithm that allows to calculate osteoporotic fracture probability based on assessing bone fracture risk factors knowing only BMI value.

**Results:**

The conducted analysis based on U Mann-Whitney test revealed that mean 10-year MOFP was significantly higher (*p* = 0.00) in women than in men. Mean 10-year MOFP in females was 4.8% (SD = 3.95%) and in males 3.21% (SD = 2.35%). Mean 10-year HOFP in women was 1.35% (SD = 2.07%) and was significantly higher (*p* = 0.03) than in men – 0.79% (SD = 1.18%).MOFP is significantly higher in patients with moderate and severe periodontitis than in those with mild periodontitis. Significant difference between MIC values and MOFP (*p* = 0.00) and HOFP (*p* = 0.00) was found. Osteoporotic fracture probability was significantly higher in patients with MIC stages C2 and C3 than C1.

**Conclusions:**

The FRAX BMI tool with radiological evaluation of periodontal disease severity and MIC index may be used in dental practice in determining individual risk of osteoporotic fracture in females and provide new opportunities of selecting those potentially more prone to such fractures.

**Trial registration:**

The approval of the local bioethics committee was obtained (KE-0254/107/2017).

## Background

According to the World Health Organization osteoporosis is defined as “a disease characterized by low bone mass and microarchitectural deterioration of bone tissue, leading to enhanced bone fragility and a consequent increase in fracture risk” [1].

Irrespective of osteoporosis type, in all its forms we deal withdisturbed bone metabolism expressed in the predominance of resorption processes over bone formation. Moreover, the characteristic features of this disease areincreased loss of bone mass and the disturbance of microdamage reparative process. Consequently, it leads to a higher risk of osteoporotic fractures [2]. The radiological image consists of decreasing quantityof trabecular bone which becomesthinner. Osteoporotic lesions also concern the cortical part of the bone [3].

Osteoporosis is a global issue, about 200 million women worldwide suffer from the disease, with the highest prevalencein North America and Europe. In Poland about 32% of women in the pre- and perimenopausal age are diagnosed with osteoporosis [4]. In 2010 it was estimated that approximately 168.000 new fragility fractures were sustained in Poland, comprising 28.000 hip fractures, 26.000 vertebral fractures, 28.000 forearm fractures and 85.000 other fractures (i.e. fractures of the pelvis, rib, humerus, tibia, fibula, clavicle, scapula, sternum and otherfemoral fractures). Over 3,5 million osteoporotic fractures each year are registered in Europe, of which 620 thousand concern hip fractures. In comparison, in the USA there are about 2 million cases of osteoporotic fractures each year, of which 300 thousand are hip fractures [5, 6]. It is predicted that more and more peoplewill be affected by osteoporosis in the futureand, consequently, the rate of hip osteoporotic fracture will increase [7]. It is caused by the fact that initially this disease develops without any symptoms, remains undiagnosed due to scarce symptomatology and itsfirst manifestationis very often a low-energy fracture of long bones or vertebrae [8].

The basic diagnostic tool in osteoporosis is the evaluation of bone mineral density (BMD) with the use of Dual Energy X-ray Absorptiometry (DXA) [9, 10]. This method is very complex, expensive and not available for all patients. Therefore, in 2008 Kanis et al. [11] proposed a unique algorithm which allows to calculate fracture probability with the use of the Fracture Risk Assessment tool (FRAX). This algorithm provides estimation of the osteoporotic fracture probability based on assessing bone fracture risk factors with or without bone mineral density value. On its basis, it is possible to assess theoccurrenceof osteoporotic hip fracture or other low-energy bone fracture in the next 10 years (absolute risk assessment –AR) [11].

Absolute Risk (AR) is evaluated on the basis of the population risk (PR) which is calculated for a specific population based on the occurrence of fractures in the prospective studies and on the value of the relative risk (RR) estimated on the basis of specific, known risk factors.

The calculation is differentfor patients with secondary osteoporosis, for whom the fracture promoting factor depending on BMD is the main disease leading to osteoporosis. The group of diseases includes: untreated hypogonadism in men and women, nonspecific inflammatory bowel diseases, extended immobilization (after spinal cord traumas,in Parkinson’s disease, muscular dystrophy), diabetes type 1 and thyroid disease (mainly untreated hyperthyroidism) [12].

By the year 2016the FRAX calculator has been adapted to calculate the risk of osteoporotic fracture in 58 countries, while the risk assessment has been divided into two types [13].

The 10-year majorosteoporotic fracture probability – (MOFP) including clinical spine, forearm, proximal humerus and 10-year hip osteoporotic fracture probability – HOFP were distinguished respectively [9]. In Poland, the interpretation of the FRAX result is as follows: low fracture risk for FRAX valuesof or below5%, medium fracture risk for FRAX valuesbetween 5 and 10% and the high fracture risk for FRAX values over 10% [14].

An increasing number of scientists are focused on examining the correlation between periodontal disease and osteoporosis. The data analysis reveals higher tendency to the loss of the alveolar bone in patients with osteoporosis [15].

Due to the fact that with the use of panoramic radiographs it is possible torecognize the features of osteoporotic process, scientists started to introduce quantitative and qualitative indices, which could help in selection ofpatients from risk groups and allow to refer them to an appropriate clinic forfurther diagnosis.

## Methods

So far, the majority of studies dealt with the comparison of BMD values measured by means of DXA with radiomorphometric measurements, but none of them described the comparison of FRAX BMI tool with panoramic measurements. Therefore, the aim of this study was to examine the correlation between radiological signs of chronic periodontitis, Mandibular Inferior Cortex (MIC) index with osteoporotic fracture probability estimated on the basis of FRAX BMI tool.

The material comprised 422 patients aged 40–89 (mean 56.14), 270 females and 152 males. The study was prospective and all patients were informed about its purpose and planned procedures then signed an informed consent. The approval of the local bioethics committee was obtained (KE-0254/107/2017).

Inclusion criteria were: individuals of both genders, aged between 40 and 90 years, with clinical diagnosis of gingivitis or periodontitis, referred for panoramic radiographydue to clinical indications. Exclusion criteria were bisphosphonate and glucocorticoids therapy, a history of rheumatoid arthritis and low quality of panoramic radiography influencing evaluation of dental and skeletal status. Extremely rare diseases with very low prevalencein the Polish population like b-thalassemia were not taken into account [16].

The research consisted of analysis of panoramic radiographs and a diagnostic survey performed in patients, necessary to use FRAX BMI tool.

All panoramic radiographs were taken by means of the VistaPano (DuerrDental, *Bietigheim*-Bissingen, Germany) and Planmeca Proscan (Helsinki, Finland) in the Dental and Maxillofacial Radiology Department of the Medical University of Lublin, Poland.

The severity of chronic periodontitis wasevaluatedin panoramic X-rays using the following radiological criteria for periodontal bone loss:

- Healthy (0) - no bone loss, i.e. there was a normal distance between the crestal bone margin and the cemento-enamel junction (CEJ) not exceeding 2-3 mm; the interdental crestal bone continuous with lamina dura of the adjacent teeth; thin even width to the mesial and distal periodontal ligament spaces; cancellous bone of the interdental crestal bone similar to the bone in periapical region.
- Mild periodontitis (1) - loss of the corticated interdental crestal margin; horizontal bone loss up to 1/3 of the alveolar bone or 1/4–1/3 of the root; local osteoporosis.
- Moderate chronic periodontitis (2) - horizontal bone loss up to 2/3 of the alveolar bone or 1/2 of the root; vertical bone defects.
- Severe chronic periodontitis (3) - horizontal bone loss over 2/3 of the alveolar bone or 1/2 of the root; multiple vertical bone defects with deep bone pockets; severe bone loss involving the tooth apex; mobility of teeth resulting in widening of periodontal ligament space; massive furcation involvement.

**Fig. 1:**
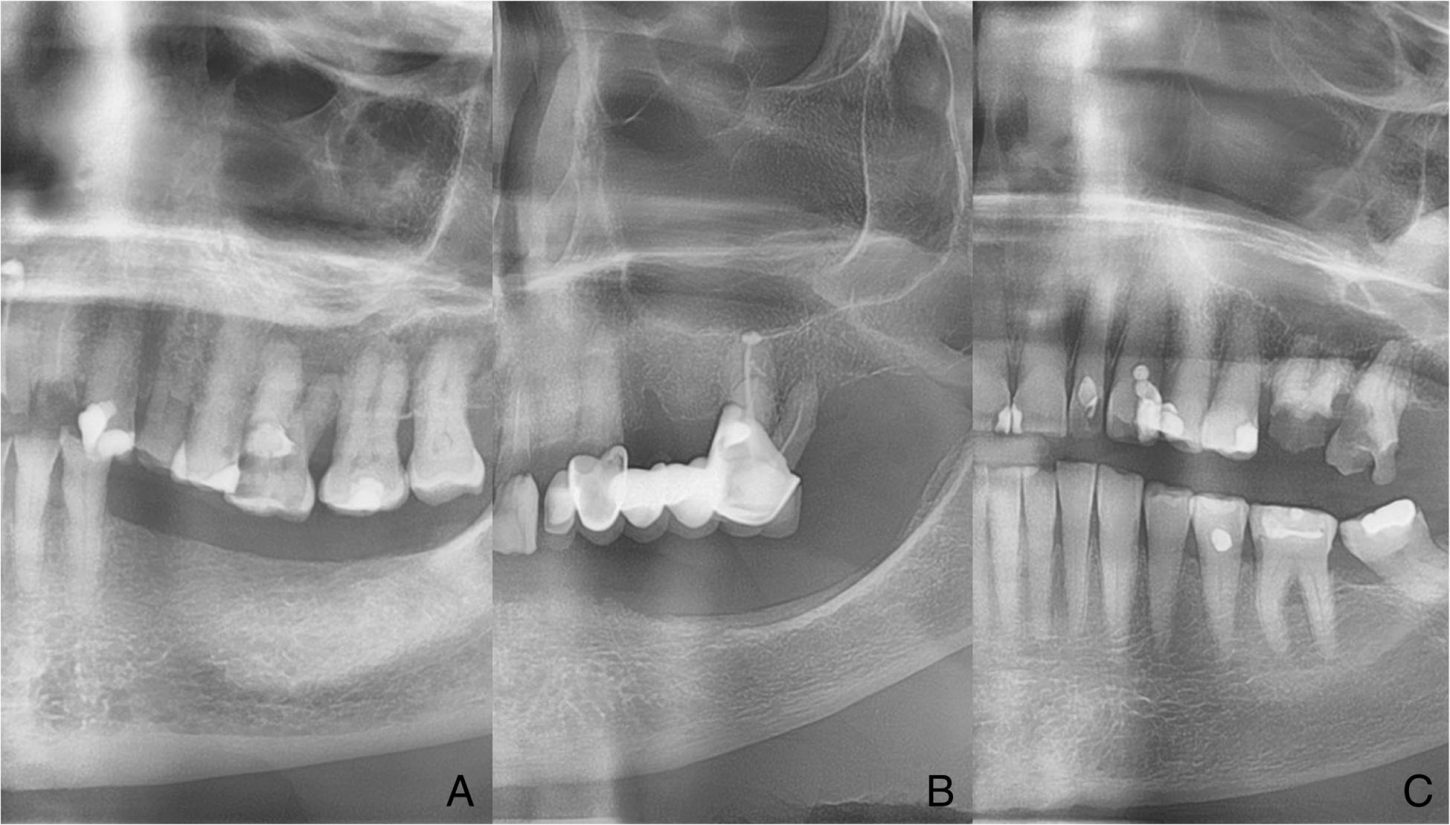
Classification of inferior mandibular cortex according to Klemetti MIC stages 1–3

The mandibular inferior cortex (MIC) status was evaluated distally from the mental foramen and divided into one of three groups according to Klemetti et al. [17], where C1 - the endosteal cortical margin is even and sharp on both sides, normal cortex; C2 - the endosteal margin has semi-lunar defects (lacunar resorption) or endosteal cortical residues on one or both sides, mild to moderate cortex erosion; C3 - the cortical layer forms heavy endosteal cortical residues and is clearly porous, severely eroded cortex (Fig. 1).

All radiographic evaluations were performed by one observer (MP). In case of problems in assigning patients to categories, second reading was performed (IRK) in order to reach consensus.

A diagnostic survey was conducted to appraise the 10-year major and hip osteoporotic fracture probability by means of FRAX BMI tool (Polish version 3.3) - an algorithm that allowed calculating the probability of osteoporotic fracture based on assessing bone fracture risk factors without bone mineral density value, knowing only body mass index (BMI) value.

**Fig. 2:**
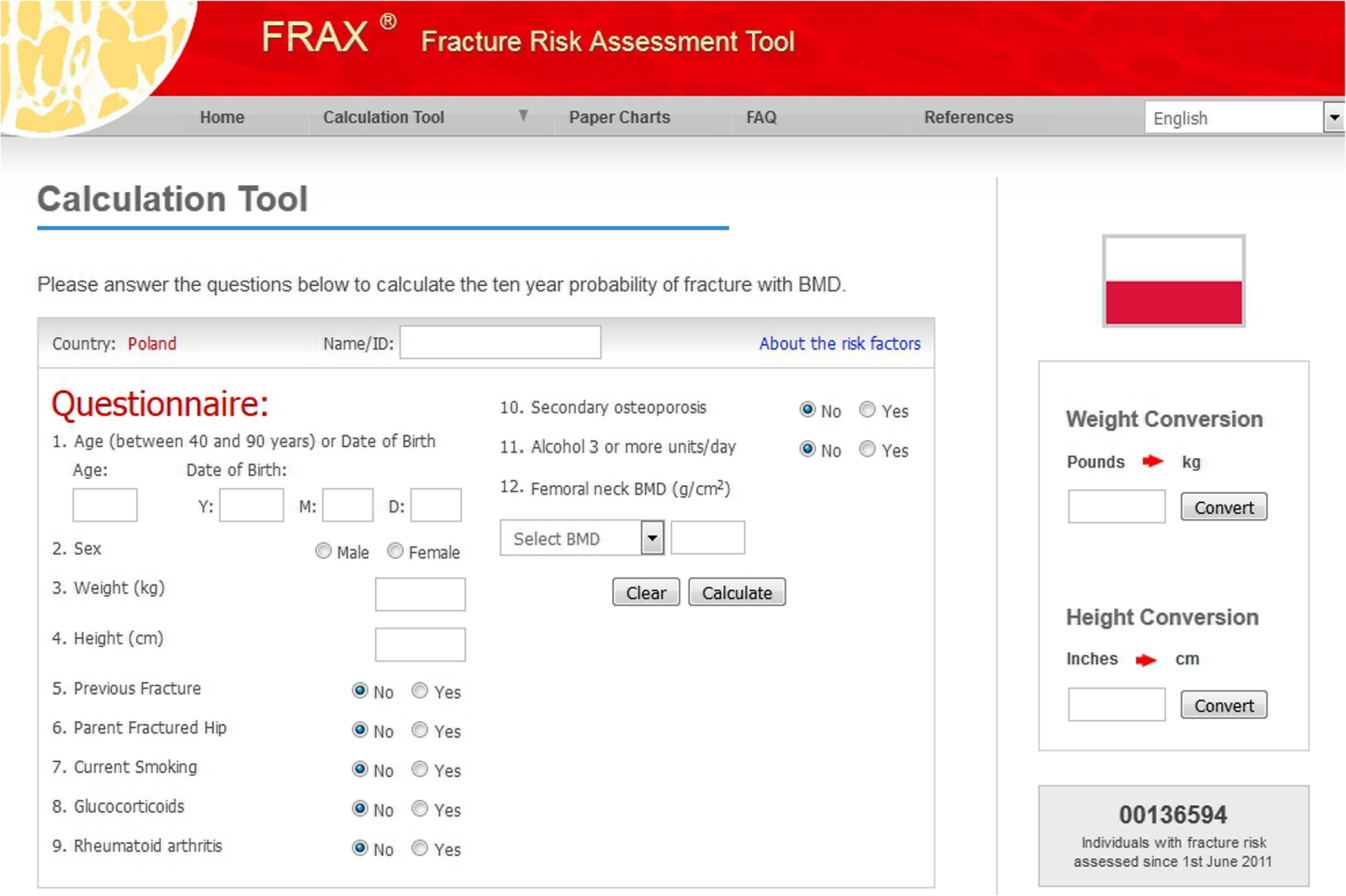
Screenshot of FRAX BMI tool – Polish version (https://www.sheffield.ac.uk/FRAX/tool.aspx?lang=en)

When calculating the above mentioned probabilities, the “calculationtool” was used, available on the website of the University of Sheffield, UK (Fig. 2) [14]. The following variables obtained as information from patients’ questionnaires were taken into account in the evaluation of the osteoporotic fracture probability:to calculate BMI (BMI = weight (kg) / height (m^2^)) - age, gender, weight in kg, height in cm;cases of bone fractures in mature life, hip fracturesin patient’s mother or father, smoking at present or in the past, consumption of 3 or more units of alcohol per day, taking glucocorticoids at present or in the past for more than three months, presence of diseasessuch as rheumatoid arthritis, diabetes type I, osteogenesis imperfecta, hyperthyroidism left untreated for a long time, hypogonadism, early menopause (before the age of 45), chronic malnutrition, malabsorption and chronic liver disease. The calculator includes a box for input of BMD value, but it is not mandatory for calculation of osteoporosis risk and the box is left blank, if BMD is not available like in the present study.

The statistical analysis was carried out using Statistica 9.1 software (StatSoft, Poland) and U Mann-Whitney as well as Kruskall-Wallis ANOVA rank tests were applied as normal distribution of data was not observed. The significance level was set at ≤0.05.

## Results

Number of patients according to gender in stages of periodontal bone loss and MIC stages are presented in the Table 1.

**Table 1:** Number of patients according to stages of periodontal bone loss and MIC stages taking into account gender

	Females	Males
Stage of periodontal bone loss		
0	39	22
1	81	44
2	96	47
3	54	39
MIC stage		
1	108	87
2	56	27
3	106	38

Based on the U Mann-Whitneytest we found that the group of women and men was homogeneous in terms of age (*p* = 0.34). The conducted analysis based on UMann-Whitney test revealed that mean 10-year MOFP was significantly higher (*p* = 0.00) in women than in men. Mean 10-year MOFPin females was 4.8% (SD = 3.95%) and in males 3.21% (SD = 2.35%). Mean 10-year HOFP in women was 1.35% (SD = 2.07%) and was significantly higher (*p* = 0.03) than in men – 0.79% (SD = 1.18%) Table 2.

**Table 2:** 10-year major osteoporotic fracture (MOFP) and hip osteoporotic fracture probability (HOFP) assessed by means of the FRAX BMI tool in female and male patients

Variable	Mean Females	SD Females	Mean Males	SD Males	*p*
MOFP	4.8	3.95	3.21	2.35	0.00
HOFP	1.35	2.07	0.79	1.18	0.03

On the basis of the Kruskall-Wallis test it was found that in group of men there was no correlation between the stage of periodontal disease and 10-year MOFP (*p* = 0.7085) as well as 10-year HOFP (*p* = 0.1831). In male patients the conducted analysis also did notreveal any significant difference between the stadium of resorption of mandibular inferior cortex according to Klemmetti (MIC index) and 10-year MOFP,as well as HOFP (*p* = 0.548, *p* = 0.7354, respectively).

In female patients we found significant differences between the stage of chronic periodontitis and 10-year MOFP (*p* = 0.0003) (Table 3) as well as 10-year HOFP (*p* = 0.00) (Table 4). The significant differenceespeciallyconcern patients from group 1(mild periodontitis) with group 2 (moderate periodontitis) and group 3 (severeperiodontitis). The 10-year MOFP was significant, higher in female patients with moderate periodontitis and severe periodontitis than with mild one. The statistical analysis including 10-year HOFPrevealed significant difference between female patients with mild periodontitis, (group 1) with moderate periodontitis (group 2) and severe periodontitis (group 3).10- year HOFP was significantly higher in female patients with moderate and severe periodontitits than in those with mild one.

**Table 3:** 10-year major osteoporotic fracture probability (MOFP) and severity of chronic periodontitis and MIC of female and male patients

Gender	Variable	Stage	Mean 10-year MOFP probability	H	p	Significance of differences (pairwise comparison)
Female	Presence and severity of chronic periodontitis	0	4.46	H = 18.50	0.0003	
		1	3.65			1–2 (*p* = 0.00)
		2	5.31			1–3 (*p* = 0.00)
		3	5.90			
	MIC	C1	3.22	H = 44.10	0.00	1–2 (*p* = 0.00)
		C2	5.36			1–3 (*p* = 0.00)
		C3	6.75			
Male	Presence and severity of chronic periodontitis	0	2.82	H = 1.387	0.7085	1–2 (*p* > 0.05)
		1	3.58			
		2	3.11			1–3 (*p* > 0.05)
		3	3.14			
	MIC	C1	2.97			1–2 (*p* > 0.05)
		C2	3.50	H = 1.208	0.548	1–3 (*p* > 0.05)
		C3	2.61

**Table 4:** 10-year hip osteoporotic fracture probability (HOFP) assessed by means of the FRAX BMI, severity of chronic periodontitis and MIC stage in female and male patients

Gender	Variable	Stage	Mean 10-year hip osteoporotic fractures probability	H	p	Significance of differences (pairwise comparison)
Female	Presence and severity of chronic periodontitis	0	1.24	H = 13.77	0.0032	1–2 (p = 0.00)
		1	0.89			
		2	1.38			1–3 (p = 0.00)
		3	2.06			
	MIC	C1	0.55	H = 52.64	0.00	1–2 (*p* = 0.00)
		C2	1.61			1–3 (*p* = 0.00)
		C3	2.37			
Male	Presence and severity of chronic periodontitis	0	0.61	H = 4.85	0.1831	1–2 (p > 0.05)
		1	0.95			
		2	0.68			1–3 (p > 0.05)
		3	0.83			
	MIC	C1	0.67	H = 0.61	0.7354	1–2 (p > 0.05)
		C2	0.92			1–3 (p > 0.05)
		C3	0.53			

Moreover, Kruskall-Wallis test revealed that in the group of women there wasa significant difference between the MIC values and 10-year MOFP (*p* = 0.00) as well as 10-year HOFP (*p* = 0.00). Significant differences were found regarding the comparison of patients with C1 stage with patients representing stages C2 and C3. 10-year MOFP was significantlyhigher in womenpresenting stages C2 and C3 of MIC index than with stadium C1.

The statistical analysis referring to 10-year HOFP revealed that there was a significant difference between female patients with stadium C1 and stadium C2 of MIC index with stadium C3. Female patients with C2 and C3 represent higher 10-year HOFP than those at stage C1.

## Discussion

Panoramic examination, a form of focal plane tomography producing a two-dimensional image, is commonly used in dental practice, but not free from disadvantages [18]. Although panoramic examination is not the main tool in diagnosing osteoporosis, there are some attempts to use it in evaluating hard tissues of mandible with reference to osteoporosis. Scientists stated that this kind of examination could be very useful in diagnosing osteoporosis and even selecting patients who need additional examination such as DXA for confirmation of diagnosis [19].

The correlation between osteoporosis and the oral health remains a subject of numerous controversies. It is important to confirm whether there is a relationship between osteoporosis and bone loss in the oral cavity.

Among others, Dervis [20] claimed that osteoporosis contributes to the loss of hard tissues supporting teeth and even loss of teeth. The study of Bałczewska [21] reported more recessions in females with lower BMD. In addition, the same author noticed more recessions in the mandible, which can be caused by earlier manifestation of the disease in the mandible in all diseases which have origin in generalized bone mineral deficiencies. Since both osteoporosis and periodontitis are caused by common factors, it is recommended to refer females (before menopause) with periodontitis for DXA examination [22, 23].

There are only a few studies taking up this subject. In a previously conducted study on the Polish population, it was revealed that there was a statistically significant correlation at all stages of chronic periodontitis and the status of inferior cortex of the mandible with a horizontal bone loss. Patients with the signs of resorption in inferior cortex (C2 and C3) had more advanced periodontal bone loss comparing to patients with healthy inferior cortex (C1) [24].

The discussion on the correlation between periodontal diseases and osteoporosis has a long history [25]. In our own research we have demonstrated, that the risk of low-energy bone fracture, which is directly a result of osteoporosis, increases with the advancing periodontal disease. The obtained results are compatible with those of other authors in this subject [15, 24, 25].

Moreover, it was proved by some authors that qualitative indexes are more useful than quantitative when evaluating the risk of osteoporosis in panoramic radiographs [26–28]. For example, Horner, Devlin, Božič and Hren confirmed in their studies the advantage of MIC over the PMI (Panoramic Mandibular Index) [27, 28]. However, so far there are not sufficient studies into the correlation between the risk calculated based on FRAX tool with the radiological image of mandible. There are some authors whose results agree with our study, but their research was based on DXA examination [29, 30]. They claimed that MIC is a good index in identifying postmenopausal patients who suffer from osteoporosis, it allows to distinguish between healthy and osteoporotic females and indicates the group of patients who need to be referred for a DXA examination [29–32]. Similarly, Božič and Hren [28] confirmed the usefulness of MIC index in osteoporosis detection.

The results of the quoted authors are in compliance with our own research in which we have revealed that the risk of osteoporosis increases with the higher stage of the resorption of the mandibular inferior cortex according to Klemetti (MIC).

The limitation of the study is lack of information on BMD values in the examined group. However, FRAX tool can be used without this value, when it is not available.

The FRAX BMI tool with radiological evaluation of periodontal disease severity and MIC index may be used in dental practice in determining individual risk of osteoporotic fracture but only in females and provide new opportunities of selecting female patients potentially more prone to such fractures.

Apart from discussed qualitive index (MIC) and periodontal bone loss in relation to FRAX BMI tool, which were the subject of this study, there are also other indices repeatedly evaluated on panoramic radiographs and accessed in correlation to decreased bone mineral density.

So far, thanks to the simplicity and low cost of examination the pantomographs were most often used to assess the thickness and integrity of the inferior cortexof mandible (IC), as well as they allowed to calculate the height of the alveolar bone (H) or the distance from the lower border of mental foramen to the lower part of inferior cortex of the mandible (h). Among all radiomorphometric indices, the biggest attention was paid to linear indicators, such as: Panoramic Mandibular Index (PMI), Mandibular Ratio (MR), Inferior Cortex (IC index) and angular, Mandibular Angle (MA).

In many research it has been repeatedly claimed that the PMI allows to identify patients who should be referred for a BMD examination. These studies confirmed significant differences between PMI values of healthy and osteoporotic patients [26, 27, 30, 33, 34]. Calciolari et al. [33] in their meta-analysis regarding the assessment of radiomorphometric indicators in pantomographic images focused more precisely on PMI. They claimed that patients with reduced mineral density had PMI ≤ 0.3. Yashoda Devi et al. [35] based research on a group of older women and observed that patients should be referred for further diagnostics towards osteoporosis only when the PMI was< 0.25. On the other hand, there are some studies which do not confirm the usefulness of PMI, because authors did not found any correlation between the lower PMI values and the decreased bone mineral density calculated with the use of DXA tool [17, 26, 28].

Another indicator, Mandibular Ratio (MR) informs about the resorption of the residual alveolar portion of the mandible. Its usefulness in detecting osteoporosis has been evaluated several times. However, most reports refer to the lack of correlation between decreased MR values and bone mineral density measured with the use of DXA method [36]. In studies of Drozdzowska et al. [36] and Ortman et al. [37] only the presence of correlation between the index value and the age of patients was proved. According to Calciolari et al. [33] assessment of the thickness of inferior cortex (IC) can be used to exclude patients at high risk with reduced bone mineral density. According to these authors, 90% of patients with value bigger than 4 mm have normal bone mineral density. Many publications confirmed that people with osteoporosis characterize significantly reduced thickness of mandibular inferior cortex [17, 38–41].

There are also scientists who are concern about angular measurements - Mandibular Angle (MA). Çakur et al. [42] have shown that values of MA decrease in men who have osteoporosis. It allows to state that is reasonable to use angular measurements to qualify patients for further diagnostics for osteoporosis. Analogous results were presented by the authors of the report, who also included women in their study group. The results showed clear differences between the values of the angle of the jaw in healthy patients, osteopenia and osteoporosis [43].

All elaborated facts stand in favour of the expediency of using this imaging method for detecting patients with decreased bone mineral density.

## Conclusions

1. The higher stage of resorption of mandibular inferior cortex according to Klemetti (MIC index), the higher 10-year MOFP and HOFP of female patients.
2. There were statistically significant differences between severity of chronic periodontitis and 10-year MOFP and HOFP in female patients.
3. The higher stage of periodontal disease, the higher osteoporotic fracture probability.

## Abbreviations

BMD: Bone mineral density
BMI: Body Mass Index
CEJ: Cemento-enamel junction
DXA: Dual Energy X-ray Absorptiometry
FRAX BMD: Fracture Risk Assessment Tool based on BoneMineral Density
FRAX BMI: Fracture risk assessment tool based on body mass index
FRAX: Fracture Risk Assessment Tool
HOFP: 10-year Hip osteoporotic fracture probability
IC: Inferior Cortex
MA: Mandibular Angle
MIC: Mandibular Inferior Cortex
MOFP: 10-year Major osteoporotic fracture probability
MR: Mandibular Ratio
PMI: Panoramic Mandibular Index
RB: Absolute Risk – AR
RP: Population risk
RW: Relative risk
WHO: World Health Organization
